# Uncovering potential single nucleotide polymorphisms, copy number variations and related signaling pathways in primary Sjogren’s syndrome

**DOI:** 10.1080/21655979.2021.2000245

**Published:** 2021-11-24

**Authors:** Xuan Qi, Xi-Qin Wang, Lu Jin, Li-Xia Gao, Hui-Fang Guo

**Affiliations:** a Department of Rheumatism and Immunology, The Second Hospital of Hebei Medical University, Shijiazhuang, Hebei, China; b Internal Medicine, Yuhua Yunfang Integrated Traditional Chinese and Western Medicine Clinic, Shijiazhuang, Hebei, China

**Keywords:** Primary Sjogren’s syndrome, whole-exome sequencing, single nucleotide variants, copy number variations, signaling pathway, Sanger sequencing, in vitro validation

## Abstract

Primary Sjogren’s syndrome (pSS) is a complex systemic autoimmune disease, which is difficult to accurately diagnose due to symptom diversity in patients, especially at earlier stages. We tried to find potential single nucleotide polymorphisms (SNPs), copy number variations (CNVs) and related signaling pathways. Genomic DNA was extracted from peripheral blood of 12 individuals (7 individuals from 3 pSS pedigrees and 5 sporadic cases) for whole-exome sequencing (WES) analysis. SNPs and CNVs were identified, followed by functional annotation of genes with SNPs and CNVs. Gene expression profile (involving 64 normal controls and 166 cases) was downloaded from the Gene Expression Omnibus database (GEO) dataset for differentially expression analysis. Sanger sequencing and in vitro validation was used to validate the identified SNPs and differentially expressed genes, respectively. A total of 5 SNPs were identified in both pedigrees and sporadic cases, such as FES, PPM1J, and TRAPPC9. A total of 3402 and 19 CNVs were identified in pedigrees and sporadic cases, respectively. Fifty-one differentially expressed genes were associated with immunity, such as BATF3, LAP3, BATF2, PARP9, and IL15RA. AMPK signaling pathway and cell adhesion molecules (CAMs) were the most significantly enriched signaling pathways of identified SNPs. Identified CNVs were associated with systemic lupus erythematosus, mineral absorption, and HTLV-I infection. IL2-STAT5 signaling, interferon-gamma response, and interferon-alpha response were significantly enriched immune related signaling pathways of identified differentially expressed genes. In conclusion, our study found some potential SNPs, CNVs, and related signaling pathways, which could be useful in understanding the pathological mechanism of pSS.

## Introduction

Primary Sjogren’s syndrome (pSS), a chronic and autoimmune condition characterized by exocrine gland dysfunction, leads to substantial morbidity. Clinically, women are affected at a rate of approximately 10 times more than men [1,2]. The disease can occur alone as pSS or be related to other connective diseases (called secondary SS), such as rheumatoid arthritis or systemic lupus erythematosus. pSS primarily affects the salivary and lacrimal grands with resultant dryness of the mouth and eyes. In pSS, fatigue is a dominant feature [3]. In addition, the presence of auto-antibodies (anti-Ro/SS-A and La/SS-B antibodies) is used for the diagnosis of pSS patients along with exocrine hypofunction [4]. It is found that some pSS patients exhibit extraglandular manifestations that affect many organs, including the liver, skin (purpura and xerosis), lungs (bronchiectasis and obstructive airway disease), joints (arthritis, myalgias and arthralgias), kidneys and nervous system, which contributing to the high burden of illness and mortality [5–7].

Generally, innate and adaptive immune responses are contributed to SS, possibly triggered by hormonal factors and viral infections in a genetically susceptible host. The pathogenesis of pSS also involves genetic elements [8,9] and subsequent environmental factors [10]. Clinically, there is no cure or effective treatment for pSS, with management of the disease based on the relief of symptoms. Provisional diagnosis is usually based on serological markers, such as anti-Ro/SSA [11] and anti-La/SSB [12]. However, these auto-antibody markers lack the sensitivity and specificity for a confirmatory diagnosis [13], making diagnosis a significant clinical challenge. Therefore, understanding the pathological mechanism of pSS is needed.

In previous studies, several single nucleotide polymorphisms (SNPs) were found in pSS, including interferon regulatory factor 5 (IRF5), family with sequence similarity 167 member A (FAM167A), signal transducer and activator of transcription 4 (STAT4), TNF alpha induced protein 3 (TNFAIP3) and TNF receptor superfamily member 13 C (TNFRSF13C) [14–21]. In this study, we tried to find potential SNPs, copy number variations (CNVs), differentially expressed genes in pSS by both WES analysis and differentially expression analysis. Sanger sequencing and in vitro validation was respectively used to validate identified SNPs and differentially expressed genes. Our study may be useful in understanding the potential pathological mechanism of pSS.

## Materials and methods

### Subjects

**Figure 1.**
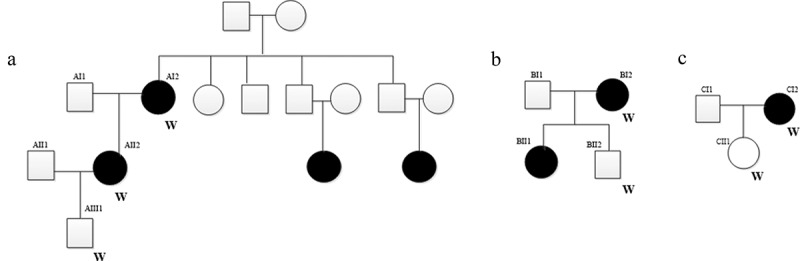
Three pSS pedigrees examined in this study

In this study, the inclusion criteria of pSS patients were as follows: (1) patients were diagnosed with pSS according to the European Standard (2002 version); (2) patients had detailed clinical and follow-up information. The exclusion criteria of patients were as follows: (1) patients with secondary SS (presents with other autoimmune diseases or in a patient previously diagnosed with a different autoimmune condition); (2) patients received radiation treatment of the head and neck before; (3) patients were infected with hepatitis C; (4) patients with acquired immunodeficiency disease (AIDS); (5) patients with preexisting lymphoma sarcoidosis; (6) Patients took anticholinergic drugs (time shorter than 4 times the half-life of the drug). There was no significant gender/age difference between patients and healthy controls (patients’ families). Finally, three pSS pedigrees (involved seven individuals) (Figure 1) and five sporadic cases were enrolled. In pedigree A, for AI2 patient, teeth flaky loss is observed for 10 years. Dry mouth, dry eyes (for 10 years), hyperglobulinemia, mumps and peripheral neuropathy were major clinical features of AII2 patient. In pedigree B, BI2 patient is characterized as dry mouth and dry eyes (2 years). In pedigree C, both lower limbs purpura for 7 years and hyperglobulinemia were major clinical features of CI2 patient. Detailed clinical information of above 12 patients is shown in Table 1. In above three pSS pedigrees, the peripheral blood samples from AI2, AII2, AIII1, BI2, BII2, CI2, CII1 individuals and 5 additional sporadic cases (D1-5) were collected for whole-exome sequencing (WES) analysis. This study was approved by the ethics committee of the Second Hospital of Hebei Medical University (2021-R305). Written consent was obtained from enrolled individuals.

**Table 1.** Clinical information of enrolled 12 individuals in the WES analysis

Patient	Age	Clinical features	Complications	Family history	Ro/La antibodies	Levels of immunoglobulin G (g/L)
AII2	50	Dry mouth, dry eyes (for 10 years), hyperglobulinemia, mumps and peripheral neuropathy	Thyroid nodules and peripheral neuropathy	Yes	Positive for SSA and SSB	29.1
AI2	70	Teeth flaky loss for 10 years	None	Yes	Positive for SSA and SSB	19
AIII1	28	None	None	Yes		
BI2	58	Dry mouth and dry eyes (for 2 years)	Hyperthyroidism	Yes	Positive for SSA and SSB	15.2
BII2	30	None	None	Yes		
CI2	36	Both lower limbs purpura for 7 years and hyperglobulinemia	None	No	Positive for SSA and SSB	22.4
CII1	9	None	None	No		
D1	75	Dry mouth and flaky tooth loss (over 20 years)	Pulmonary interstitial fibrosis with infection	No	Positive for SSA	14.1
D2	49	Teeth flaky loss (for 10 years) and dry mouth and dry eyes (for 5 years)	Liver cirrhosis	No	Positive for SSA and SSB	33.8
D3	31	Dry mouth (for 2 years) and the whole body fatigue (for 0.5 year)	Renal tubular acidosis	No	Positive for SSA and SSB	36.3
D4	70	Teeth flake loss (for 8 years) and dry mouth	Thrombocytopenia	No	Positive for SSA and SSB	16.4
D5	63	Repeated intrahepatic calculi for 14 years	Intrahepatic bile duct stones	No	Negative for SSA and SSB	26

A, B, and C represent individual in the pedigrees; D represents individual in the sporadic cases.

The box and circle represent male and female, respectively. The black color represents individual with pSS. A, B, and Crepresent pedigrees 1, 2, and 3, respectively. W: individual that enrolled for WES analysis.

### Exome capture and sequencing

Genomic DNA was extracted from peripheral blood and used for quality inspection and library construction. One percent agarose gel was used to test the integrity and purity of the DNA sample. The DNA concentration was determined by fluorescence quantitation or enzyme label analyzer. 1 μg genomic DNA was ultrasonically interrupted to 250–300 bp using the Covaris instrument. After the interruption, the DNA was firstly electrophoretically detected with agarose gel, and then purified using AxyPrep MAG PCR clean up kit. The terminal repair reaction system was formulated and purified after a suitable temperature reaction period. The terminal reaction system with ‘A’ and the joint reaction system was prepared. The pre-polymerase chain reaction (PCR) reaction system was prepared and purified by AxyPrep MAG PCR clean up kit. A certain amount of PCR products (1 µL) were hybridized and eluted by Agilent Sureselect Hybridization and Wash kit. The product after hybridization elution was amplified and purified using AxyPrep MAG PCR clean up kit. The quality and yield of constructed library were respectively tested using Agilent 2100 BioAnalyzer and ABI Steponeplus Real-Time PCR System. The qualified library was sequenced on the Xten platform. The original image data obtained by sequencing was transformed into sequence data by base calling. The data was divided into clean data and adaptor data after index and basic data quality control.

### Identification of SNPs and CNVs in pSS

In order to obtain high-quality data, the original sequencing data was firstly filtered by fastp software [22]. The specific steps were as follows: the adapter sequence was deleted from reads; the bases containing non-AGCT at the 5 ‘end was deleted before shearing; the ends of reads with low sequencing quality (<Q20) were trimmed; 10% reads containing N were removed; small segments with length less than 25 bp were deleted. Burrows-Wheeler Aligner (BWA) software (http://bio-bwa.sourceforge.net/) [23] was applied to match the sequenced fragments back to the reference genome of hg38. Genome Analysis Toolkit (GATK4) software [23] was utilized to remove the sequences generated by PCR-duplication. Strelka software (https://github.com/Illumina/strelka) [24] was devoted for mutation detection of SNP and indel. Annotate Variation (ANNOVAR) [25] and tool for assessment and prioritization in exome studies (tapes) software [26] were used for annotation of mutation sites, including annotation of gene structure of mutation sites, annotation of genome characteristics, prediction of non-synonymous mutations, annotation of known mutation database, annotation of mutation-related gene function. Mutations were annotated with dbSNP ID and mutation frequencies of 1000 genomes, and evaluated the effects on diseases by combining SIFT, PloyPhen, MutationAssessor and LRT methods. The mutations were first identified. Then rare possible pathogenic mutations were screened (mutation sites with mutation frequency ≥0.05 in 1000genome, EXAC and ESP6500 databases were screened out). Synonym mutations and mutation sites located in the intron region were also screened out. Finally, those sites with American Society for Medical Genetics and Genomics (ACMG) scoring for ‘pathogenic’, ‘likely pathogenic’ and ‘variants of uncertain significance (VUS)’ were identified. Exomecopy software [27] was used to filter parameters of copy.count and nranges. The filtering criterion was that parameter copy.count ≠ 2 and nranges >5. Then, cnvscan software [28] was applied to annotate the CNVs, including important functional information, known CNVs and clinical information. The annotation databases included GENCODE, PhastCon, Sanger high-resolution CNVs, DGV, Curated high-quality DGV, 1000 Genomes CNVs, OMIM, and Clinvar. Firstly, CNVs were obtained. Then, strict screening was performed through the annotation information including CNV prediction score >40, CNVQ (median quality score of CNVs) >40 and not reported in CNV data set. In order to improve the accuracy of the results, only those genes in the CNVs region were retained.

### Functional annotation and protein–protein interaction (PPI) analysis of mutation genes

To study the biological function of identified mutation genes, Gene Ontology (GO) and the Kyoto Encyclopedia of Genes and Genomes (KEGG) pathway enrichment analysis was performed via DAVID software [29]. P value < 0.05 was the threshold of significantly enriched GO and KEGG terms. Additionally, the String tool was utilized to perform PPI analysis on the proteins encoded by mutated genes.

### Identification and functional analysis of differentially expressed genes in GEO dataset

In order to further investigate the molecular mechanism and provide more evidence for screening pathogenic mutations in pSS, we searched transcriptome data of pSS from the GEO database with keywords of (Sjogren’s syndrome) AND ‘Homo sapiens’[porgn:__txid9606]. Finally, a total of 3 GEO datasets of microarray gene expression (GSE66795, GSE145065, and GSE84844, involving whole blood or peripheral blood samples from 64 normal controls and 166 cases) was obtained. The difference analysis was performed by metaMA and limma packets under the screening criteria of p value <0.05. All gene expression matrices of filtered datasets were enriched and analyzed by GSEA 4.1.0 software [30]. GSEA analysis allows customization of gene sets. The ‘h.all.v7.2.symbols.gmt’ is the.gmt file format is required for analysis based on personalized requirements. P value < 0.05 was selected as the significantly enriched gene sets. In addition, Reactome was also used for functional analysis of genes. Significantly enriched signaling pathways were identified under the screening criteria of P value < 0.05.

### Variant validation

Sanger sequencing was devoted to validate the candidate gene mutations. The template DNA, primers and Extender PRC-to-Gel Master Mix were melted the on ice for PCR reaction. The PCR reaction condition was as follows: degeneration at 95°C for 5 min, 95°C for 30s, 67°C for 30s, 72°C for 1 min of 14 cycles, extension at 72°C for 7 min. PCR products (5 µL) were detected and purified by agarose gel electrophoresis and enzymolysis approach, respectively. The qualified PCR products were purified by enzymatic hydrolysis. PCR products were sequenced in the ABI 3730XL by using sequencing kit of BigDye. The reference sequences NM_001278508.4 of ZNF180, NM_003890.2 of FCGBP and NM_001124759.5 of FRG2C were used.

### In vitro validation of differentially expressed genes

To investigate the expression of candidate mutated genes in pSS, qPCR was performed. Peripheral blood samples of 13 individuals (8 individuals in 3 pedigrees and 5 sporadic cases) were collected. Total RNA of the peripheral blood samples was extracted and synthesized DNA by FastKing cDNA first strand synthesis kit (TIANGEN). Then, real-time PCR was performed in the SuperReal PreMix Plus (SYBR Green) (TIANGEN). GAPDH and ACTB were used for internal reference. Relative gene expression was analyzed by fold change method. The two-sample unpaired T test was used for statistical analysis. P < 0.05 represents statistical significance.

## Results

In this study, we aimed to find the potential SNPs, CNVs, and related signaling pathways in the peripheral blood of 12 individuals with pSS. In addition, gene expression profile was downloaded from the GEO dataset to identify differentially expressed genes in pSS. Finally, Sanger sequencing and in vitro validation was applied to validate the identified SNVs and differentially expressed genes in pSS.

### Identification of candidate SNPs and related functional analysis in pedigrees

**Figure 2.**
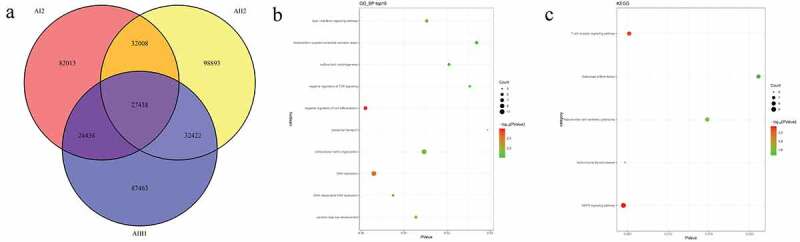
Number and functional analysis of mutant genes in pedigree A

**Figure 3.**
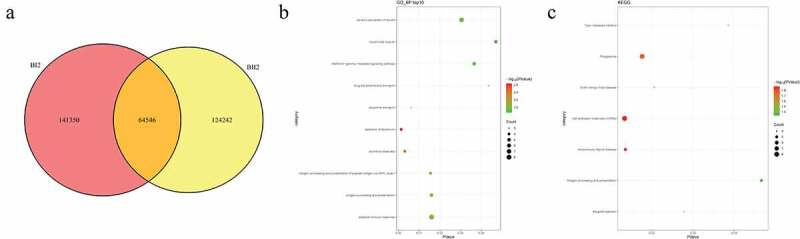
Number and functional analysis of mutant genes in pedigree B

**Figure 4.**
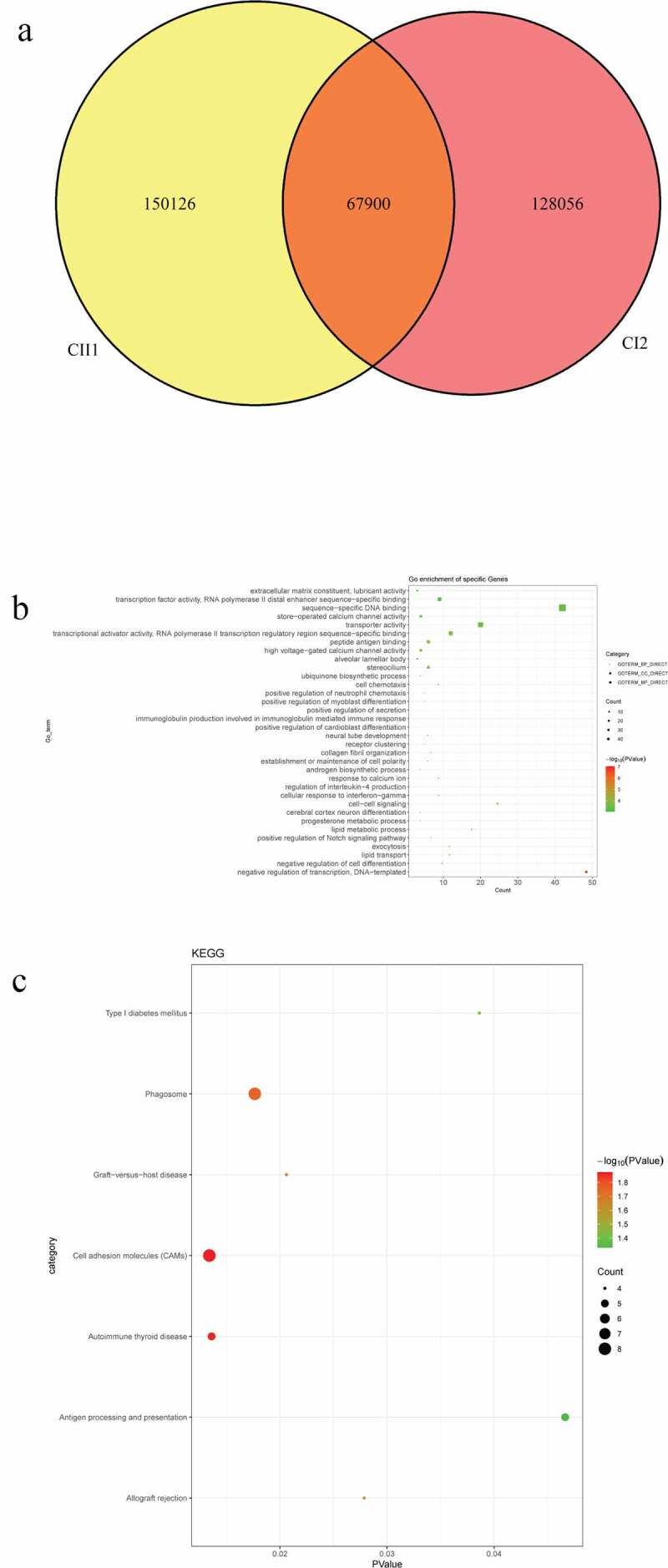
Number and functional analysis of mutant genes in pedigree C

In pedigree A, a total of 32,008 mutations were firstly screened (Figure 2(a)). These mutations were presented in both AI2 and AII2 patients, but not in AII1 individual. After further screening for rare possible pathogenic mutations, 728 SNPs and Indel of 605 genes and 688 SNPs and Indel of 566 genes were identified in AI2 patient andAII2 patient, respectively. Among which, a total of 559 common mutant genes were identified between AI2 patient and AII2 patient. In the GO and KEGG analysis of these common mutant genes, negative regulation of cell differentiation (Figure 2(b)) and AMPK signaling pathway (Figure 2(c)) was the most significantly enriched biological process and signaling pathway, respectively. In pedigree B, a total of 141,350 mutations were firstly screened (Figure 3(a)). These mutations were presented in BI2 patient, but not in BII2 individual. After further screening for rare possible pathogenic mutations, 384 SNPs and Indel of 373 genes were identified in BI2 patient. In the GO and KEGG analysis of above 373 mutant genes, detection of bacterium (Figure 3(b)) and cell adhesion molecules (CAMs) (Figure 3(c)) was the most significantly enriched biological process and signaling pathway, respectively. In pedigree C, a total of 128,056 mutations were firstly screened (Figure 4(a)). These mutations were presented in CI2 patient, but not in CII1 individual. After further screening for rare possible pathogenic mutations, 1880 SNPs and Indel of 1517 genes were identified in CI2 patient. In the GO and KEGG analysis of above 1517 mutant genes, negative regulation of transcription, DNA-templated (Figure 4(b)) and Cell adhesion molecules (CAMs) (Figure 4(c)) was the most significantly enriched GO terms and signaling pathway, respectively. It is worth mentioning that a total of 1177 common mutations were identified between pedigree A, B, and C. These common mutations covered 15 mutation genes (Table 2), such as zinc finger protein 180 (ZNF180) and chromosome 18 open reading frame 25 (C18orf25), which located in the exon region. No signaling pathway was enriched by these 15 mutation genes.

**Table 2.** Common mutation genes between pedigree A, B, and C

Gene	Start_end	Func.refGene	AI2	AII2	BI2	CI2
SSU72	1541728_1541728	UTR3	G→A	G→A	G→A	G→A
SSU72	1541943_1541945	UTR3	GCA→G	GCA→G	GCA→G	GCA→G
LOC105370401	22483099_22483099	Upstream	A→G	A→G	A→G	A→G
ELP4\PAX6	31789577_31789577	UTR3	T→TA	T→TA	T→TA	T→TA
HLA-DRB1	32578815_32578815	UTR3	A→G	A→G	A→G	A→G
TAF1L	32635707_32635708	Upstream	CT→C	CT→C	CT→C	/
ZNF180	44477666_44477666	Exonic	G→C	G→C	G→C	G→C
ZNF180	44479350_44479350	Exonic	G→C	G→C	G→C	G→C
C18orf25	46253735_46253738	Exonic	TCTG→T	TCTG→T	TCTG→T	TCTG→T
KCNB1	49483100_49483100	Upstream	C→CT	/		/
DDX28	68021475_68021475	UTR3	C→T	C→T	C→T	C→T
FOXC2	86569037_86569037	UTR3	C→T	C→T	C→T	C→T
CWF19 L1	100233166_100233167	UTR3	TA→T	TA→T	TA→T	TA→T
BTLA	112499472_112499472	UTR5	T→TAA	/	/	/
LOC653513	149197754_149197754	ncRNA_exonic	T→C	T→C	T→C	T→C
LOC285097	242003892_242003892	ncRNA_exonic	C→T	C→T	C→T	C→T

A: Venn diagrams of mutations between AI2, AII2, and AII1 individuals. B: top 10 significantly enriched biological processes of mutant genes. C: all significantly enriched signal pathways of mutant genes.

A: Venn diagrams of mutations between BI2 and BII2 individuals. B: top 10 significantly enriched biological processes of mutant genes. C: all significantly enriched signal pathways of mutant genes.

A: Venn diagrams of mutations between CI2 and CII1 individuals. B: all significantly enriched GO terms of mutant genes. C: all significantly enriched signal pathways of mutant genes.

### Identification of candidate SNPs and related functional analysis in sporadic cases

**Figure 5.**
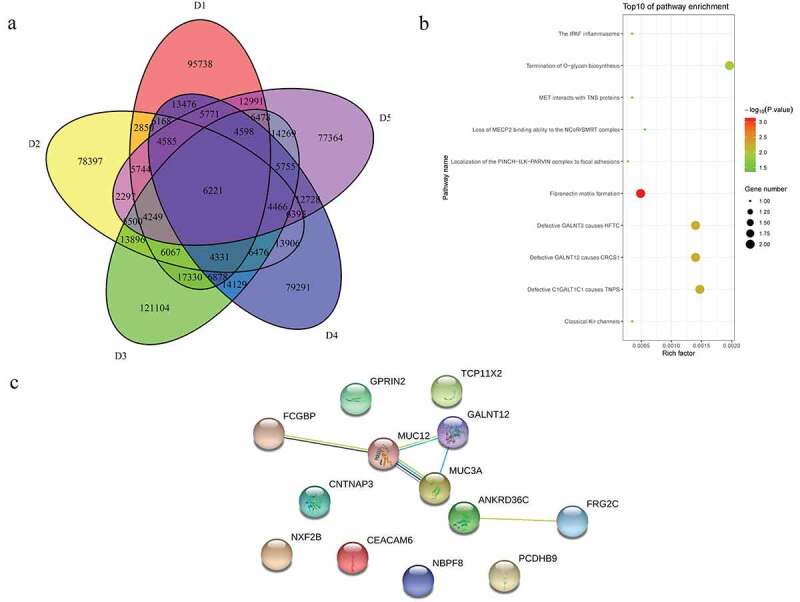
Common mutations and PPI analysis of mutant genes in 5 sporadic cases

A total of 6221 common mutations were screened in 5 sporadic cases (Figure 5(a)). After further screening for rare possible pathogenic mutations, 264 SNPs and Indel of 90 genes, 259 SNPs and Indel of 89 genes, 261 SNPs and Indel of 91 genes, 260 SNPs and Indel of 90 genes and 265 SNPs and Indel of 96 genes were identified in D1, D2, D3, D4, and D5 patient, respectively. A total of 77 common mutation genes were identified from above 5 patients. It is a pity that no signaling pathway was enriched by these 77 mutation genes in the GSEA analysis. Based on the Reactome analysis, only 10 significantly enriched signaling pathways were identified, including fibronectin matrix formation, defective GALNT3 causes HFTC, defective GALNT12 causes CRCS1, defective C1GALT1C1 causes TNPS, termination of O-glycan biosynthesis, localization of the PINCH-ILK-PARVIN complex to focal adhesions, the IPAF inflammasome, classical Kir channels, MET interacts with TNS proteins and loss of MECP2 binding ability to the NCoR/SMRT complex (Figure 5(b)). To further predict the function of the mutant genes, mutant sites located in the exon region of the gene were screened, which involved 92 mutant sites of 13 genes. PPI network was constructed based on these 13 mutation genes (Figure 5(c)). In the network, mucin 12, cell surface associated (MUC12) and mucin 3A, cell surface associated (MUC3A) were proteins encoded by mutation genes with a high degree. According to the literature, MUC family genes (such as MUC12) carry a lot of mutations in autoimmune diseases [31]. It is speculated that their mutations are not specific to the occurrence of pSS. Therefore, MUC family genes were excluded. Another three proteins encoded by mutation genes with a high degree were included, including Fc fragment of IgG binding protein (FCGBP), ankyrin repeat domain 36 C (ANKRD36C) and FSHD region gene 2 family member C (FRG2C). Detailed mutation information about FCGBP, ANKRD36C and FRG2C is shown in Table 3.

**Table 3.** Mutation information of FCGBP, ANKRD36C, and FRG2C in the sporadic cases

Gene	Start_end	Func.refGene	D1	D2	D3	D4	D5
FCGBP	39886243_39886243	Exonic	C→G	C→G	C→G	C→G	C→G
FCGBP	39886257_39886257	Exonic	C→T	C→T	C→T	C→T	C→T
FRG2C	75665673_75665674	Exonic	AG→A	AG→A	AG→A	AG→A	AG→A
FRG2C	75665948_75665948	Exonic	T→A	T→A	T→A	T→A	T→A
ANKRD36C	95950756_95950756	Exonic	C→A	C→A	C→A	C→A	C→A
ANKRD36C	95950758_95950758	Exonic	G→C	G→C	G→C	G→C	G→C

A: Venn diagrams of common mutations. B: Reactome analysis of common mutant genes. C: PPI analysis of common mutant genes.

### Screening of possible pathogenic SNPs in pSS

In order to further explore whether there are common mutation genes in family patients and sporadic patients, the common SNPs were intersected. A total of 20 common SNPs were obtained. After further screening for rare possible pathogenic mutations, a total of 5 SNPs and Indel of 5 genes were identified (Table 4), such as FES proto-oncogene, tyrosine kinase (FES), protein phosphatase, Mg^2+^/Mn^2+^ dependent 1 J (PPM1J) and trafficking protein particle complex subunit 9 (TRAPPC9).

**Table 4.** Common mutation genes in family patients and sporadic patients

Gene	Start	Position	AI2	AII2	AIII3	BI2	BII2	CI2	CII1	D1	D2	D3	D4	D5
FRG1DP\FRG2EP	29327738	Intergenic	G→A	G→A	/	G→A	/	G→A	/	G→A	G→A	G→A	G→A	G→A
FES	90891941	Intronic	G→C	G→C	/	G→C	/	G→C	/	G→C	G→C	G→C	G→C	G→C
PPM1J	112713064	Intronic	T→TT	T→TT	/	/	/	/	/	/	/	/	T→TT	/
TRAPPC9	139,971,349	Intronic	C→T	C→T	/	C→T	/	C→T	/	C→T	C→T	C→T	C→T	C→T

### Identification of candidate CNVs and related functional analysis in pedigrees and sporadic cases

**Figure 6.**
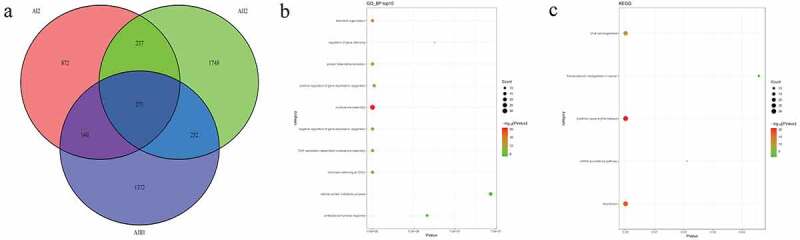
Pathogenic CNVs and functional analysis of involving genes in pedigree A

**Figure 7.**
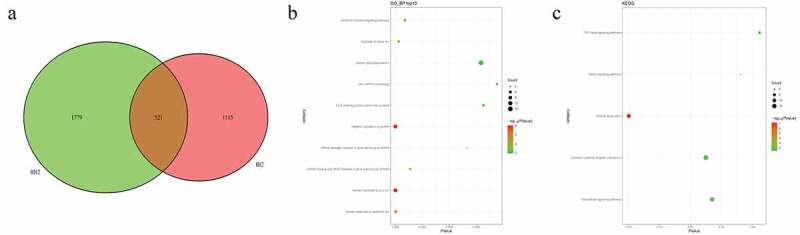
Pathogenic CNVs and functional analysis of involving genes in pedigree B

**Figure 8.**
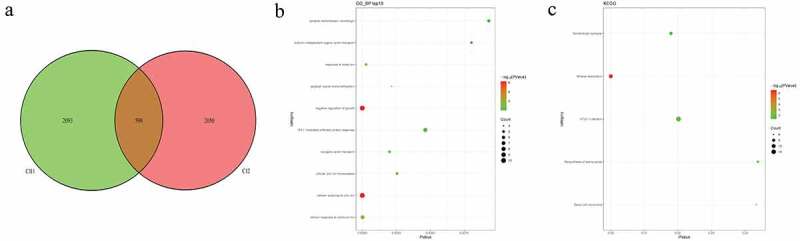
Pathogenic CNVs and functional analysis of involving genes in pedigree C

**Figure 9.**
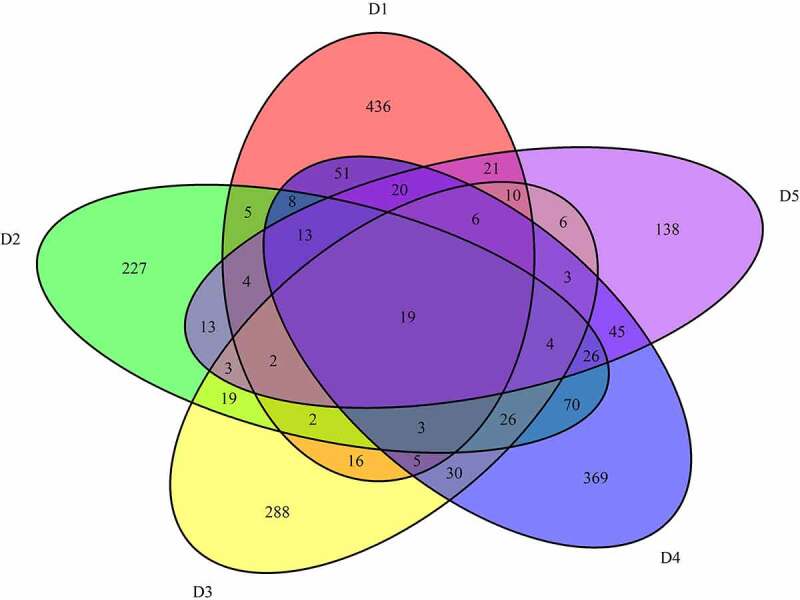
Venn diagrams of pathogenic CNVs in 5 sporadic cases

In pedigree A, a total of 237 CNVs were screened (Figure 6(a)). After further screening, 1239 pathogenic genes in above CNVs region were identified. In the GO and KEGG analysis of these pathogenic genes, nucleosome assembly (Figure 6(b)) and systemic lupus erythematosus (Figure 6(c)) was the most significantly enriched biological process and signaling pathway, respectively. Some genes in the CNV deletion regions were significantly associated with systemic lupus erythematosus (Table 5), such as tumor necrosis factor (TNF) in chromosome 6 and small RNA binding exonuclease protection factor La (SSB) in chromosome 2. In pedigree B, a total of 1115 CNVs were screened (Figure 7(a)). After further screening, 1651 pathogenic genes in above CNVs region were identified. In the GO and KEGG analysis of these pathogenic genes, cellular response to zinc ion (Figure 7(b)) and mineral absorption (Figure 7(c)) was found. It is noted that SSB gene in the CNV deletion region in chromosome 2 screened in pedigree A still existed in pedigree B (Table 6). In pedigree C, a total of 2050 CNVs were screened (Figure 8(a)). After further screening, 1901 pathogenic genes in above CNVs region were identified. In the GO and KEGG analysis of these pathogenic genes, negative regulation of growth (Figure 8(b)) and HTLV-I infection (Figure 8(c)) were found. A total of 19 common CNVs (involved 54 pathogenic genes) were identified in 5 sporadic patients (Figure 9, Supplementary Table 1).

**Table 5.** Partial pathogenic genes in the CNV deletion regions and involved systemic lupus erythematosus signaling pathway in family A

Chr	Start_end	Length	Partial genes	CNV status
6	24865529_26370831	1,505,302	HIST1H4A:F|TRIM38:F|HIST1H4G:F|HIST1H4F:F|HIST1H4C:F|HIST1H4B:F|HIST1H4H:F|LRRC16A:F|SLC17A1:F|HIST1H1D|RP11-191A15.1:F|RP11-191A15.2:F|HIST1H2BG:F|RP11-191A15.4:F|HIST1H3D:F|HIST1H3F:F|HIST1H2BE:F|U91328.22:F|U91328.21:F|U91328.20:F|RNY5P5:F	Deletion
6	31515940_31659457	143,517	GPANK1:F|BAG6:F|LY6G5C:F|LST1:F|PRRC2A:F|APOM:F|NCR3:F|UQCRHP1:F|C6orf47:F|LY6G5B:F|LTB:F|LTA:F|TNF:F|C6orf47-AS1:F|CSNK2B:F|CSNK2B-LY6G5B-1181:F| AIF1:F	Deletion
2	170462549_171258201	795,652	KLHL23:F|AC016772.4:F|PTCHD3P2:F|CCDC173:F|METTL5:F|AC012594.1:F|PPIG:P|UBR3:F|PHOSPHO2:F|RNU6-1006P:F|SSB:F	Deletion

**Table 6.** Partial pathogenic genes in the CNV deletion regions in family B

Chr	Start_end	Length	Partial genes	CNV status
2	170462549_171258201	795,652	KLHL23:F|AC016772.4:F|PTCHD3P2:F|CCDC173:F|METTL5:F|AC012594.1:F|PPIG:P|UBR3:F|PHOSPHO2:F|RNU6-1006P:F|SSB:F	Deletion
2	31515940_31659457	143,517	GPANK1:F|BAG6:F|LY6G5C:F|LST1:F|PRRC2A:F|APOM:F|NCR3:F|UQCRHP1:F|C6orf47:F|LY6G5B:F|LTB:F|LTA:F|TNF:F|C6orf47-AS1:F|CSNK2B:F|CSNK2B-LY6G5B-1181:F| AIF1:F	Deletion

A: Venn diagrams of pathogenic CNVs between AI2, AII2, and AII1 individuals. B: top 10 significantly enriched biological processes of involving genes in CNVs regions. C: all significantly enriched signal pathways of involving genes in CNVs regions.

A: Venn diagrams of pathogenic CNVs between BI2 and BII2 individuals. B: top 10 significantly enriched biological processes of involving genes in CNVs regions. C: all significantly enriched signal pathways of involving genes in CNVs regions.

A: Venn diagrams of pathogenic CNVs between CI2 and CII1 individuals. B: top 10 significantly enriched biological processes of involving genes in CNVs regions.C: all significantly enriched signal pathways of involving genes in CNVs regions.

### Identification of differentially expressed genes in GEO dataset

In order to further investigate the molecular mechanism of pSS, a total of 3 GEO datasets of microarray gene expression (GSE66795, GSE145065 and GSE84844, involving 64 normal controls and 166 cases) was obtained (Table 7). A total of 2643 differentially expressed genes (1177 up-regulated and 1466 down-regulated) were identified. For example, TNF, basic leucine zipper ATF-like transcription factor 2 (BATF2), basic leucine zipper ATF-like transcription factor 3 (BATF3), poly (ADP-ribose) polymerase family member 9 (PARP9), interleukin 15 receptor subunit alpha (IL15RA) and leucine aminopeptidase 3 (LAP3) were up-regulated in pSS. While, SSB, FES, PPM1J and TRAPPC9 were down-regulated in pSS. In addition, 51 differentially expressed genes were associated immunity. These immune related genes were compared in the CNVs regions of three pedigrees (Table 8) and five sporadic cases (Table 9). Significantly, BATF3 gene in the CNV deletion region in chromosome 1 was found in the pedigree; LAP3 gene in the CNV deletion region in chromosome 4 was found in the sporadic case. Interestingly, three genes in the CNV deletion regions were found in both pedigree and sporadic case, including BATF2 in chromosome 11, PARP9 in chromosome 3 and IL15RA in chromosome 10.

**Table 7.** 3 GEO datasets of gene expression in pSS

Dataset	Sample	Microarray analysis	Group
GSE66795	Whole blood	Illumina	Control(29)+pSS(131)
GSE145065	Peripheral blood monouclear cell	Illumina	Control(5)+pSS(5)
GSE84844	Whole blood	Affymetrix	Control(30)+pSS(30)

**Table 8.** CNVs amplification/deletion of 51 immune related genes in 3 pedigrees

Sample	Chr	Start_end	Length	Partial genes	CNV status
Pedigree A	1	211960974_213139120	1,178,146	BATF3:F|PPP2R5A:F|ATF3:F	Deletion
Pedigree B	3	122044140_122662382	618,242	DTX3L:F|PARP9:F|PARP15:F|HSPBAP1:F|EIF4BP8:F|CCDC58:F	Duplication
Pedigree B	10	5960305_6002530	42,225	FBXO18:P|IL15RA:P|RP11-536K7.3:F	Deletion
Pedigree B	10	6005706_6258743	253,037	IL2RA:F|RP11-414 H17.5:F|RP11-414H17.2:F|PFKFB3:P|RP11-536K7.5:F|RBM17:F|IL15RA:P	Deletion
Pedigree B	3	122121607_122459960	338,353	DTX3L:F|HSPBAP1:P|PARP9:F	Deletion
Pedigree C	11	64502584_64889285	386,701	SYVN1:P|CDC42BPG:F|CDCA5:F|AP000436.4:F|PYGM:F|ZNHIT2:F|BATF2:F|VPS51:F|MIR194-2:F|PPP2R5B:F	Deletion
Pedigree C	7	92252350_92821676	569,326	AC002454.1:F|SAMD9L:F|HEPACAM2:P|CDK6:P|RN7SL7P:F|SAMD9:F	Duplication

**Table 9.** CNVs amplification/deletion of 51 immune related genes in 5 sporadic cases

Sample	Chr	Start_end	Length	Partial genes	CNV status
D1	1	155721751_155932974	211,223	KIAA0907:F|ARHGEF2:P|RXFP4:F|RIT1:F|RNU4-19P:F|SYT11:F|GON4L:P|RP11-101O6.2:F	Duplication
D1	1	160616660_160769872	153,212	CD48:F|SETP9:F|LY9:P|RP11-404 F10.2:F|SLAMF7:F|SLAMF1:P	Duplication
D2	10	5959553_6008302	48,749	FBXO18:P|IL15RA:P|RP11-536K7.3:F	Deletion
D2	10	6008108_6258743	250,635	IL2RA:F|RP11-414H17.5:F|RP11-414H17.2:F|PFKFB3:P|RP11-536K7.5:F|RBM17:F|IL15RA:P	Deletion
D2	10	7327828_7622027	294,199	RNU6-535P:F|SFMBT2:P|RP5-1092K5.2:F|RP11-385N23.1:F|ITIH5:P|RP5-1031D4.2:F	Deletion
D2	11	64502584_64889285	386,701	CDCA5:F|AP000436.4:F|PYGM:F|ZNHIT2:F|BATF2:F	Deletion
D3	3	122121607_124721159	2,599,552	PARP9:F|ROPN1:F|PDIA5:F|DIRC2:F|MUC13:F|RP11-521 J5.1:F|SEMA5B:F|ADCY5:F|SEC22A:F|HSPBAP1:F|FAM162A:P|PARP14:F|	Deletion
D3	4	17510894_17830011	319,117	MED28:F|snoU13:F|QDPR:P|LAP3:F|AC006160.5:F|AC006160.4:F|NCAPG:P|CLRN2:F|FAM184B:F|DCAF16:F	Deletion
D3	8	90801549_90976735	175,186	OSGIN2:F|COX6B1P6:F|RIPK2:P|RNU6-925P:F|NBN:P	Deletion
D3	11	57317441_57461387	143,946	YPEL4:F|ZDHHC5:P|CLP1:F|UBE2L6:F|AP000662.4:F|SERPING1:F|RPS4XP13:F|SMTNL1:P	Deletion
D3	11	64502584_64889285	386,701	BATF2:F|VPS51:F|MIR194-2:F|PPP2R5B:F	Deletion
D4	3	122121607_122459960	338,353	PARP9:F|RP11-592P9.1:F|RP11-299J3.6:F|PARP15:F|WDR5B:F|KPNA1:F	Deletion
D4	10	5960305_6258743	298,438	IL2RA:F|FBXO18:P|RP11-414H17.2:F|PFKFB3:P|RP11-414H17.5:F|RP11-536K7.5:F|RBM17:F|RP11-536K7.3:F|IL15RA:F	Deletion
D4	11	64502584_64889285	386,701	|BATF2:F|VPS51:F|MIR194-2:F|PPP2R5B:F	Deletion
D4	12	113346341_113554966	208,625	DTX1:F|RP1-71H24.1:P|RP1-71H24.4:F|OAS1:P|OAS3:F|OAS2:F|RASAL1:P|RPS15AP32:F	Duplication
D5	3	122121607_122459960	338,353	PARP9:F|RP11-592P9.1:F|RP11-299J3.6:F|PARP15:F|WDR5B:F|KPNA1:F|EIF4BP8:F|RP11-299J3.8:F|PARP14:F|FAM162A:P	Deletion

### Functional analysis of differentially expressed genes in GEO dataset

**Figure 10.**
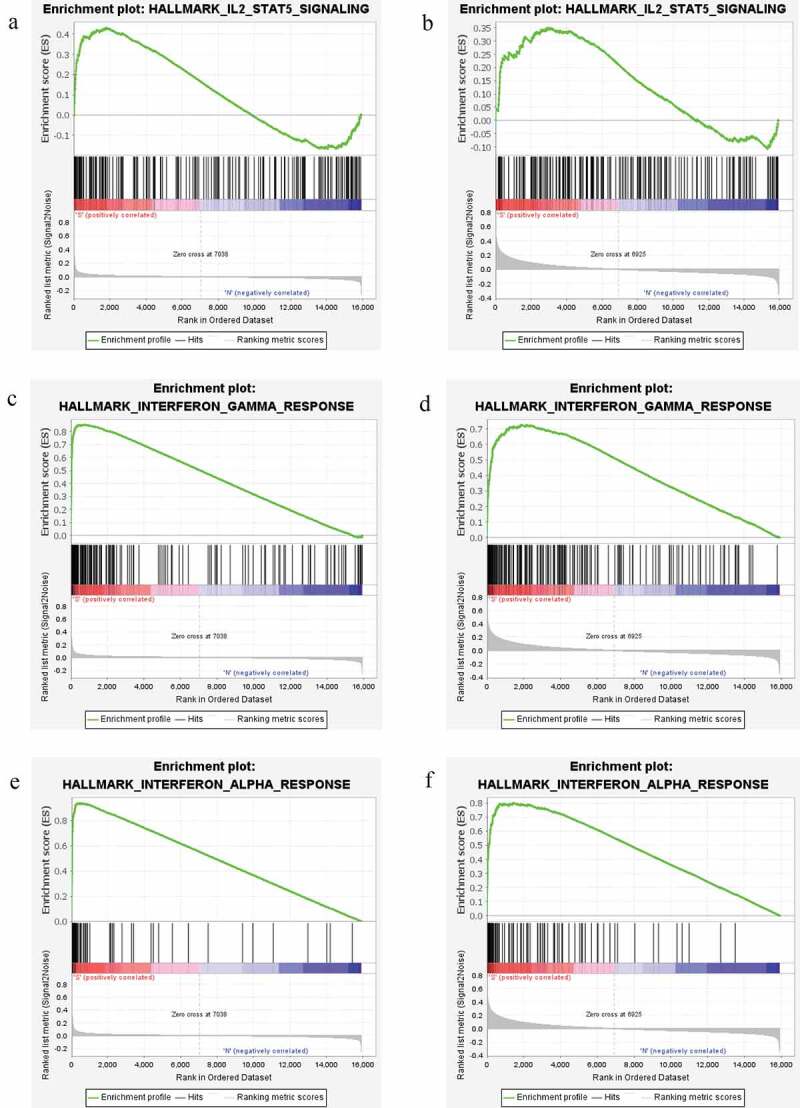
GSEA enrichment pathways of differentially expressed genes in both GSE66795 dataset and GSE84844 dataset

In order to study the potential biological function of genes, GSEA analysis was performed. The result showed that nine gene sets in GSE66795 dataset were significantly up-regulated, 22 gene sets in GSE84844 dataset were significantly up-regulated in pSS. While the enrichment result of gene sets in GSE145065 dataset was not significant. Six common significantly enriched signaling pathways were identified between GSE66795 dataset and GSE84844 dataset, including apoptosis, complement, IL2-STAT5 signaling, interferon-gamma response, inflammatory response, and interferon-alpha response. Among which, IL2-STAT5 signaling (Figure 10(a,b)), interferon-gamma response (Figure 10(c,d)) and interferon-alpha response (Figure 10(e,f)) were related to immune function.

A, C, and E respectively represent IL2-STAT5 signaling, interferon-gamma response and interferon-alpha response in GSE66795 dataset. B, D, and F respectively represent IL2-STAT5 signaling, interferon-gamma response and interferon-alpha response in GSE84844 dataset.

### Sanger sequencing

**Figure 11.**
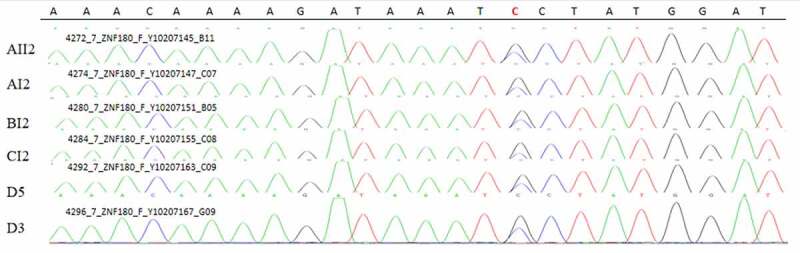
Sanger validation results of ZNF180 variant in 4 patients in 3 pedigrees and 2 sporadic patients. Red base represents the mutation site

**Figure 12.**
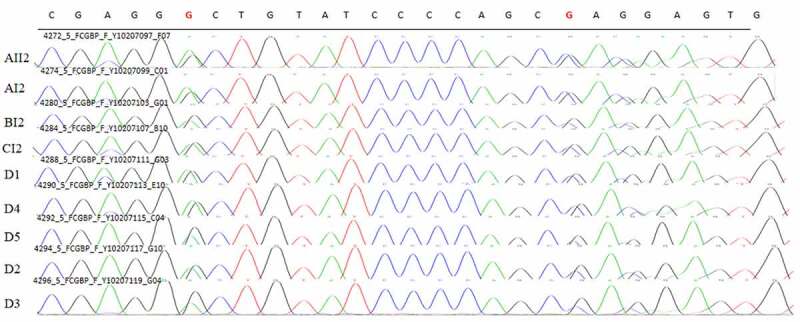
Sanger validation results of FCGBP variant in 4 patients in 3 pedigrees and 5 sporadic patients. Red base represents the mutation site

**Figure 13.**
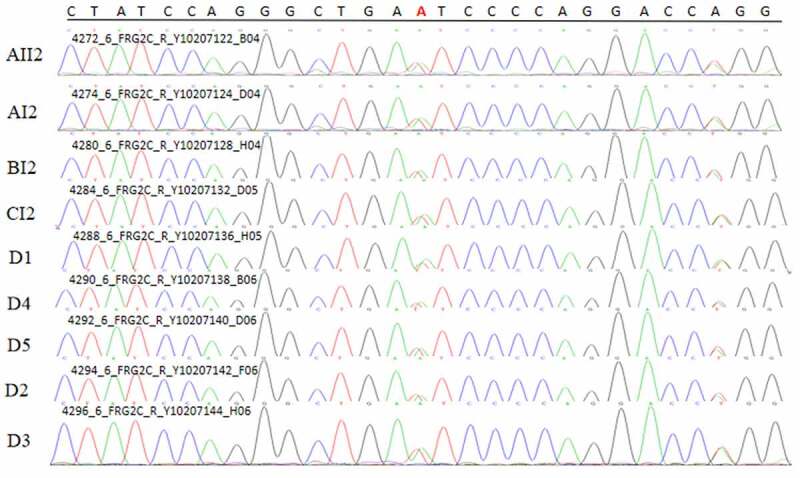
Sanger validation results of FRG2C variant in 4 patients in 3 pedigrees and 5 sporadic patients. Red base represents the mutation site

A common mutation gene in three pedigrees (ZNF180) and two common mutation genes in five sporadic cases (FCGBP and FRG2C) were selected for Sanger sequencing. The result showed that ZNF180 mutation (G > C) was found in four patients in three pedigrees. Moreover, the mutation was also found in two sporadic patients (Figure 11). In addition, FCGBP mutations (C > G and C > T) (Figure 12) and FRG2C mutation (T > A) (Figure 13) were found in five sporadic cases. Furthermore, above mutations were also found in four patients in three pedigrees. This suggested that mutations in ZNF180 (G > C), FCGBP (C > G and C > T) and FRG2C (T > A) may be associated with pathology of pSS.

### In vitro validation of differentially expressed genes

**Figure 14.**
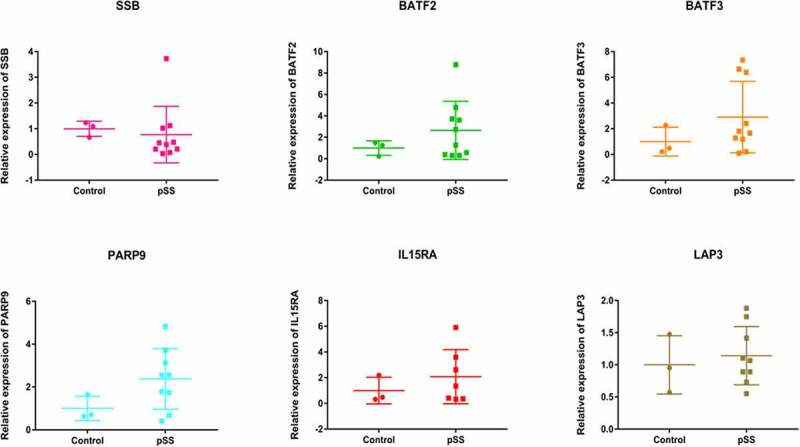
The in vitro validation of SSB, BATF2, BATF3, PARP9, IL15RA, and LAP3 in pSS

Peripheral blood samples of 13 individuals (8 individuals in 3 pedigrees and 5 sporadic cases) were collected for in vitro validation of differentially expressed genes. SSB, BATF2, BATF3, PARP9, IL15RA and LAP3 were selected (Figure 14). The result showed that BATF2, BATF3, PARP9, IL15RA and LAP3 were up-regulated, SSB was down-regulated, which was in line with the differentially expression analysis. It is noted that the CNV deletion in chromosome was found in above genes. This indicated that CNV deletion in chromosome may affect above gene expression.

## Discussion

In identification of potential SNPs in pSS, we found 15 common SNPs (such as ZNF180 and C18orf25) and 77 common SNPs (such as FCGBP, ANKRD36C and FRG2C) in 3 pedigrees and 5 sporadic cases, respectively. Significantly, a total of 5 SNPs were identified in both pedigrees and sporadic cases, such as FES, PPM1J and TRAPPC9. It is noted that SNPs in ZNF180, FCGBP and FRG2C were found in both pedigrees and sporadic cases in the Sanger sequencing. It is reported that ZNF180 is involved in immune cell infiltration in melanoma cell maintenance [32]. C18orf25 plays an important role in the lacrimal functional unit [33]. FCGBP, an immune response gene, is enriched in CD8^+^ T cells [34]. FCGBP has been regarded as a constituent of human saliva and high expressed in salivary glands and several autoimmune diseases [35]. MEV Johansson and TK Albert et al found that FCGBP bound to the Fc portion of IgG molecules during the inflammatory reaction, which enhances bacteria opsonization [36,37]. ANKRD36C is expressed on immune cells (such as lymphocytes) with ion channel inhibitory properties [38]. FES is related to immune response [39]. FES is a differentially methylated gene in systemic lupus erythematosus patients [40]. PPM1J is involved in the response of *Staphylococcus aureus* infection [41]. In addition, we found that AMPK and cell adhesion molecules (CAMs) were the most significantly enriched signaling pathways of identified SNPs in three pedigrees. Adiponectin prevents interferon (IFN)-induced apoptosis of salivary gland cells by activating AMPK signaling pathway [42], which suggests that AMPK could protect salivary gland cells from apoptosis. Metformin plays an anti-inflammatory and anti-immunomodulatory effects by activating AMPK signaling pathway. Development of drugs for SS should be taken into consideration the potential activation of AMPK signaling pathway under long-term metformin treatment [43]. In pSS, Turkcapar et al. found that some CAMs (intercellular adhesion molecule-1 and vascular cell adhesion molecule-1) were indispensable factors for glandular damage, lymphocyte recruitment and vasculitis development, which indicating the importance of CAMs pathway in pSS [44]. This suggested that above SNPs may play an important role in inflammation and immune response in the development of pSS.

In identification of potential CNVs in pSS, we found that some genes in the CNV deletion regions were significantly associated with systemic lupus erythematosus, such as TNF in chromosome 6 and SSB in chromosome 2 in pedigrees A. It is noted that SSB gene in the CNV deletion region in chromosome 2 screened in also existed in pedigree B. Furthermore, SSB was down-regulated in pSS in the differentially expression analysis, which was validated by qPCR. It has been demonstrated that some proinflammatory cytokines, such as tumor necrosis factor (TNF)-α is up-regulated in salivary gland tissue of pSS patients [45]. TNF-α also induces activation of matrix metalloproteinase-9, and leads to destruction of the salivary gland [46]. Elevated level of TNF-α is found in tears and peripheral blood of pSS patients [47,48]. SSB is an inflammatory marker. Antibody producing plasma cells with specificity for SSB are detected in the infiltrating lymphocytes of salivary glands [49]. A recent study proposed that anti-SSB antibody is a risk factor related to increased mortality in patients with pSS [50]. Additionally, we found that identified CNVs were associated with systemic lupus erythematosus, mineral absorption and HTLV-I infection. Like pSS, systemic lupus erythematosus is also an autoimmune disease. pSS patients experience far more abundant pH drops in saliva when exposed to acidic challenges, which result in a higher risk of tooth demineralization [51]. Some down-regulated genes are significantly associated with mineral absorption in pSS [52]. HTLV-I could infect salivary epithelial cells with a low capacity of destroying glandular tissue in SS [9]. This indicated that TNF and SSB may be involved in the inflammatory response in the salivary gland and tear gland in pSS patients.

In identification of differentially expressed genes in pSS, we found that some up-regulated genes were associated with immunity, such as including BATF3, LAP3, BATF2, PARP9 and IL15RA. Moreover, BATF3 gene in the CNV deletion region in chromosome 1 was found in the pedigree; LAP3 gene in the CNV deletion region in chromosome 4 was found in the sporadic case. Interestingly, three genes in the CNV deletion regions were found in both pedigree and sporadic case, including BATF2 in chromosome 11, PARP9 in chromosome 3 and IL15RA in chromosome 10. In dendritic cell subsets, BATF3 is associated with regulatory T cell induction [53,54]. BATF3-dependent cDC1s are involved in phagocytosis, contributing to protection against some bacterial, viral and fungal infections [55,56]. LAP3 is an IFN-γ associated immunity gene and involved in defense and inflammatory response [57]. BATF2 play a key role in regulating immune related cells, such as dendritic cells and T cells [58–60]. It is found that BATF2 is up-regulated genes in SS patients [61]. Batf2^−/−^ mice had more severe infection of *Trypansoma* [62]. PARP9 is involved in innate immunity and cell death [63]. In pSS, hypomethylation of PARP9 is found in the B cells in minor salivary glands [64]. IL15RA plays a regulatory role in immune and inflammation [65]. This suggested that BATF3, LAP3, BATF2, PARP9, and IL15RA could play important roles in inflammatory and immune response. In addition, CNVs changes in these genes may affect their gene expression level. In the KEGG analysis, we found that IL2-STAT5, interferon-gamma response and interferon-alpha response were significantly enriched immune related signaling pathways of identified differentially expressed genes. IL2 and downstream STAT5 are crucial to maintain homeostasis and function of immunosuppressive Tregs [66]. Interferon-gamma is critical for the development of the lacrimal gland and salivary gland [67,68]. Increased level of interferon-gamma is found in pSS patients in lacrimal, tears, conjunctiva, and salivary gland [69]. Moreover, depression, pain, and other neurological manifestations of SS are related to interferon-gamma inducible kynurenine metabolic pathway activity [70]. Up-regulated genes induced by interferon-alpha have been found in peripheral blood and minor salivary gland tissues from patients with pSS [71–73]. Thus, it can be seen that immunity may play an important role in the process of pSS.

## Conclusion

In summary, we found some potential SNPs, CNVs and related signaling pathways in pSS, which could be useful in understanding the potential pathological mechanism of pSS. However, there are some limitations of our study. Firstly, the pSS pedigree and sporadic cases were small, a larger patient cohort is necessary to validate the identification of these novel SNPs and CNVs; Secondly, the sample size in the qPCR is small, larger numbers of samples are further needed; Thirdly, the underlying molecular mechanism of SNPs and CNVs is not deeply investigated, further animal model cell experiments or is needed; Finally, some studies of immune mechanisms of pSS are further needed in our further investigation.

## Supplementary Material

Supplemental Material

## Data Availability

All data are available in the article.
